# Enduring contributions to implementation science: Honoring the legacy of Bryan R. Garner

**DOI:** 10.3389/frhs.2025.1698749

**Published:** 2025-10-23

**Authors:** Sara J. Becker, James H. Ford II, Joseph E. Glass, Heather J. Gotham, Hannah K. Knudsen, Andrew Quanbeck, Mat Roosa, Emily C. Williams

**Affiliations:** ^1^ Center for Dissemination and Implementation Science, Northwestern University Feinberg School of Medicine, Chicago, IL, United States; ^2^ Social and Administrative Sciences Division, School of Pharmacy, University of Wisconsin-Madison, Madison, WI, United States; ^3^ Chestnut Health Systems, Lighthouse Institute, Eugene, OR, United States; ^4^ Center for Dissemination and Implementation, Stanford University School of Medicine, Palo Alto, CA, United States; ^5^ Department of Behavioral Science, University of Kentucky College of Medicine, Lexington, KY, United States; ^6^ Department of Family Medicine and Health, University of Wisconsin-Madison, Madison, WI, United States; ^7^ Roosa Consulting LLC, Syracuse, NY, United States; ^8^ Department of Health Systems and Population Health, University of Washington School of Public Health, Seattle, WA, United States

**Keywords:** implementation science, design, implementation climate, turnover, in memoriam

With heavy hearts, we are writing to celebrate the scientific contributions of our colleague and friend, Bryan R. Garner, PhD, who passed away incomprehensibly at the age of 49 on June 20, 2025. Bryan was a professor at The Ohio State University, where he served as Director of Dissemination & Implementation Science in the Division of General Internal Medicine and Director of Dissemination & Implementation Research for the Center for the Advancement of Team Science, Analytics, and Systems Thinking (CATALYST). Bryan formed enduring collaborations and friendships across the institutions where he worked and trained. He maintained career-long relationships with colleagues from Texas Christian University, where he earned his PhD in Experimental Psychology (1–3), and from Chestnut Health Systems (4–7) and RTI International (8–10), where he built and expanded his research program.

Bryan was an implementation scientist to his core. As beautifully stated in his obituary, “he devoted his life's work to improving evidence-based practices within real-world settings and underserved populations, particularly individuals affected by substance use disorders and HIV.” Bryan's program of research was characterized by ambition, profound creativity, a playful curiosity, and an impatience for contributions that he viewed as slow or incremental in nature. Bryan approached his research portfolio with a seemingly limitless reservoir of energy. He was never content with the status quo and had a reputation among friends and colleagues for “MacGyvering” new scientific solutions. His contributions to the field of implementation science were far-reaching, having served as Principal Investigator of 7 R01s, core faculty in 2 center grants, and a leader within the Ohio State Clinical and Translational Science Institute. More importantly, his implementation research challenged existing paradigms, by introducing new trial designs, frameworks, pragmatic tools, and ways of thinking about vexing problems. In the following sections, we highlight five of his major contributions to his field of implementation science, before discussing his enduring impact on his teams and collaborators.

## Interrogating causes and consequences of staff turnover

As an early career researcher, Bryan posed and answered critically important questions about staff turnover. His work in this area reflected his ability to do more with less, by building off existing national initiatives to forge new lines of inquiry. In the early 2000s, most staff turnover studies were cross-sectional in nature and little was known about antecedents of turnover and the effects of turnover on patient outcomes. Bryan capitalized upon a SAMHSA-funded demonstration project (11), which included data from 3,021 adolescent patients, 208 therapists, and 34 organizations, to obtain an R01 titled, *Impact, Predictors, and Mediators of Therapist Turnover*. His programmatic research culminated in several major contributions to the literature on staff turnover.

First, Bryan documented that 31% of substance use therapists and 19% of leaders turned over on an annual basis, rates that were consistent with turnover in other industries (12). Second, he found that staff attaining competence in evidence-based practice delivery generally remained committed to their organization, with those providers who attained competence having annual turnover rates of less than 5% (13). Third, he identified an array of multi-level, significant predictors and mediators of turnover including organizational functioning, job satisfaction, psychological climate, and burnout (14, 15). Fourth, he demonstrated that turnover intentions were dynamic and that *change* in factors like job satisfaction and role clarity predicted turnover intentions (15). And finally, he documented that staff turnover was not consistently associated with adverse adolescent outcomes as expected and, in some cases, was associated with more positive outcomes (12, 16): these findings challenged prevailing assumptions and suggested that turnover could present an opportunity for positive “realignment” of the team. Across this portfolio, Bryan documented multiple “firsts” in the SUD treatment field while making substantive contributions to the broader industrial-organizational psychology literature on turnover (17).

## Evaluating multi-level implementation strategies targeting implementation climate

After demonstrating that antecedents of staff turnover were multi-level, Bryan developed a decades-long commitment to championing the design, development, refinement and evaluation of multi-level, theory-driven implementation strategies. His work was heavily informed by the theory of implementation climate: the idea that for implementation to be successful, the innovation must be expected, supported, and rewarded (18). To target implementation climate, Bryan frequently deployed multi-level strategies containing both incentivization and facilitation. He posited that facilitation addressed the expected and supported elements of implementation climate, whereas incentivization addressed the rewarded element of implementation climate. His work in this area was characterized by multiple innovations.

In the facilitation domain, Bryan developed, evaluated, and widely disseminated the Implementation and Sustainment Facilitation Strategy (ISF) strategy. The ISF strategy is rooted in principles of motivational interviewing and contains a series of facilitator-led exercises designed to help implementation teams anticipate and address common implementation challenges. One of Bryan's R01s, the *Substance Abuse Treatment 2 HIV Care (SAT2HIV) trial*, tested the effectiveness of the ISF strategy as an adjunct to the real-world strategy used by the Addiction Technology Transfer Centers (ATTCs; didactic workshop + performance feedback + consultation) across 39 HIV service organizations (19, 20). The primary outcome analysis demonstrated that adding the ISF strategy to the ATTC strategy significantly improved implementation effectiveness (defined as the quality and consistency of implementation) and decreased the odds of clients using their primary substance after intervention exposure (21). In a follow-up analysis, Bryan's team showed that adding the ISF to the ATTC strategy was cost effective (22).

Related to the incentivization domain, Bryan was among the first investigators to employ pay-for-performance (P4P) as an implementation strategy in behavioral health. His first R01, the *Reinforcing Therapist Performance* project, interrogated whether P4P could improve therapists' intention to deliver high quality treatment with a specific focus on the delivery of the Adolescent Community Reinforcement Approach (A-CRA) for adolescents with substance use disorders. This project efficiently capitalized on existing infrastructure in the field, recruiting and randomizing ninety therapists from 32 SAMHSA funded grants that were all receiving training in A-CRA to training as usual or training as usual plus P4P. Results of this trial provided evidence of both the effectiveness and cost-effectiveness of P4P as an implementation strategy (23–25).

After establishing that ISF and P4P were each effective in isolation, Bryan led a series of studies testing multi-level strategies that integrated ISF and P4P. As a follow-up to the SAT2HIV project, he led a hybrid type 3 trial—aptly called *SAT2HIV II*—across 30 new HIV service organizations that attempted to improve upon the results observed in SAT2HIV by comparing ATTC + ISF vs. ATTC + ISF + P4P. His results demonstrated that the model combining ISF and P4P increased the number of motivational interviews implemented (26). He also co-led *Project MIMIC (Maximizing Implementation of Motivational Incentives in Clinics)* (27), another hybrid type 3 trial across 28 opioid treatment programs. Project MIMIC demonstrated that an implementation strategy combining ISF and P4P that incorporated stakeholder preferences (28, 29) was associated with superior quality and consistency of evidence-based practice (i.e., contingency management) delivery as well as superior rates of patient abstinence, relative to the standard ATTC strategy (30, 31).

Individually, each of Bryan's 4 R01s testing multi-level implementation strategies was ambitious due to the number of partner organizations and systematic examination of theory-driven research questions. When combined, his 4 R01s testing multi-level implementation strategies generated a robust pattern of results across 125 + organizations—spanning adolescent treatment, opioid treatment programs, and HIV service settings—that strategies targeting implementation climate are associated with superior implementation and patient outcomes.

## Elucidating how organizations make decisions about what to implement

Bryan's work studying multi-level implementation strategies focused on the Preparation, Implementation, and Sustainment phases of the EPIS continuum (32). In the latter years of his career, Bryan systematically examined how real-world organizations made decisions about what to implement during the Exploration phase. He led an R01 titled *Identifying and Disseminating Substance, Treatment, and Strategy Recommendations to AIDS Service Organizations (STS4HIV)*, which aimed to empirically identify stakeholder-driven recommendations for improving substance use service integration within HIV service organizations**.** Within 9 months of receiving the notice of grant award, Bryan launched one of the largest, most rapid, and most inclusive Delphi survey processes in the substance use treatment field. First, to understand which substance use disorders have the greatest negative impact on people with HIV in the U.S, Bryan conducted a “Stakeholder-Engaged Real-Time Delphi” (SE-RTD) survey with 643 stakeholders across multiple groups (clients and staff of HIV service organizations, and HIV/AIDS Planning Council members) (33). Results revealed that alcohol, methamphetamine, and opioid use disorders were perceived as most critical to address. Bryan and his team subsequently conducted two additional SE-RTDs. The first engaged 202 HIV service organizations to elucidate which substance use interventions were perceived as the best fit within their settings, and found that motivational interviewing, cognitive behavioral therapy, and buprenorphine were favored (34). The second involved 64 AIDS Education and Training Network Centers to evaluate the fit of potential implementation strategies and revealed that disseminating information had the best setting-strategy fit (35).

Bryan harnessed the SE-RTD approach to support the efficient sharing of information and to reduce between-group differences resulting from lack of information, knowledge, and/or understanding to enable more rapid consensus. His innovative, systematic use of SE-RTD furthered prior efforts to improve care for substance use disorders within HIV treatment settings by eliciting timely, actionable information from the stakeholders themselves.

## Generating novel study designs

Given his penchant for research projects that defied the status quo, it is not surprising that Bryan often found conventional research designs lacking. As was true to his nature, he didn't bemoan the lack of methods or try to shoehorn his questions into standard paradigms—with his characteristic grin and limitless energy, he simply generated his own designs.

The methods employed in Bryan's experimental studies were widely recognized for their ambition and creativity. Common in the implementation science field are single randomized controlled trials that contain a dual emphasis on effectiveness and implementation, often leading to compromises in design or statistical power. Bryan upended this prevailing approach in his SAT2HIV trial by launching two distinct randomized controlled trials in a single study (19, 20). One of the experiments focused on clinical effectiveness (19), and the other on implementation effectiveness (20). Experimental psychologists like Bryan often conduct two distinct experimental trials in a single study in a highly controlled laboratory setting with convenience samples of college students. In contrast, Bryan used this dual-experiment design in a multisite cluster-randomized hybrid type-2 effectiveness implementation trial across 39 HIV service organizations spanning 23 U.S. states, with each experiment representing a distinct Specific Aim. This trial expanded assumptions of what is feasible in implementation research and is featured in Landes and colleagues overview of hybrid effectiveness-implementation trials as a “rarer” example of a “dual-randomized” hybrid type 2 design (36).

Bryan subsequently bridged his interest in hybrid designs (employed in the *Reinforcing Therapist Performance, SAT2HIV, SAT2HIV II, and Project MIMIC* trials) with his interest in eliciting stakeholder feedback (employed in the *Identifying and Disseminating Substance, Treatment, and Strategy Recommendations to AIDS Service Organizations* trial) to advance the concept of stakeholder-engaged hybrid trial designs. He codified a novel approach to comprehensively study dissemination, implementation, effectiveness, sustainment, economics, and level-of-scaling (DIeSEL) under the umbrella of one study (37). The DIeSEL hybrid design is a phased approach that begins with engaging community and organizational stakeholders who might consider using a new evidence-based practice and ends by helping them scale the practice throughout their organization and community. The first step of a DIeSEL design is to conduct a dissemination experiment to test strategies for getting organizations to commit to adopting an evidence-based practice. Organizations who choose to adopt are then randomized to a second experiment to determine if an implementation facilitation strategy improves the consistency and quality of implementation. The same experiment is further leveraged to determine whether the facilitation strategy impacts client-level changes in substance use, and whether facilitation improves staff retention and staff-level sustainment of the evidence-based practice. Next, the DIeSEL design incorporates analyses to determine if the implementation strategy was cost-effective for payers, and to evaluate the extent that the practice was taken to scale throughout the organizations.

Bryan proposed the DIeSEL design to improve the efficiency of implementation trials by unifying intervention effectiveness and implementation strategies within a single trial design and further expanding to include dissemination, economic analysis, sustainment, and scaling. Similar to the Stakeholder-Engaged Real-Time Delphi, the design reflected Bryan's commitment to engaging community partners in the implementation process from the get-go. Bryan's creativity and commitment to his community partners resulted in a highly innovative design. If adopted, DIeSEL has the potential to revolutionize the conduct of hybrid designs by incorporating elements of partner engagement, sequential experimentation, economic relevance, and multi-level outcome analysis.

## Creating pragmatic tools and measures to make implementation science accessible

Similar to the way Bryan generated novel methods to engage stakeholders, he created pragmatic tools to make the field of implementation science more welcoming for non-specialists. As core faculty within several NIH-funded Centers, he sought to develop user-friendly, “off-the-shelf” tools and measures to make complex implementation science constructs accessible for all.

Through the NIDA-funded Center for Dissemination and Implementation At Stanford (C-DIAS) and HEAL Data2Action Research Adoption Support Center (RASC), Bryan led a national workgroup to develop the Strategies Timeline, Activities, and Resources (STAR) Log (https://www.c-dias.org/implementation-guides-and-measures/). The STAR Log is a management tool to clearly document the use of implementation strategies in research studies, enabling analysis of study effects and replication of results to spread and scale effective interventions. This work led to Bryan developing two further tools, the Strategies Timeline, Activities, and Rationale (STARationale) Table to justify choice of implementation strategies, and the Strategies Timeline, Activities, and Resources (STAResources) Table to document resources needed for replication (38). Bryan also developed brief, highly accessible measures based on theory, including innovation-values fit to understand staff perceptions about how interventions match their values (39), setting-intervention fit to measure appropriateness of treatment interventions for clinical/service settings (34), and setting-strategy fit to measure appropriateness of implementation strategies to be delivered by intermediary-purveyor organizations (35).

In addition to being an innovative and big-thinker scientist, Bryan was also a clear and generous communicator of science. He innovated and then disseminated his innovations in engaging, inviting, and whipsmart scientific presentations and prolific writing. Bryan was not interested in creating things that were proprietary; by contrast, he believed strongly that science was a means of addressing disparities [see (40, 41)] and should be available to all. Bryan's commitment to sharing his work was reflected his presentations and peer-reviewed publications, which often contained links to detailed manuals and how-to guides for the resources he created [see (20, 35)], as well as actionable tips and tricks (42–44).

One salient example of Bryan's commitment to disseminating his ideas and his methods was his published protocol for a scoping review on priority aims and testable hypotheses (PATH) in implementation research. In this protocol (45), Bryan defined and clearly articulated four clear knowledge gaps in the implementation research field, outlined three testable hypotheses, and specified his plans to conduct a scoping review and create an evidence map documenting progress towards testing these hypotheses. While it has become increasingly common to pre-register scoping reviews in Open Science Framework, the level of detail with which Bryan outlined his thoughts about the state of the field and the specificity of his ambitious research agenda were exceedingly rare. His protocol was cited in a pre-mortem about the field of implementation science (46) and was sufficiently detailed to enable several of his collaborators to take forward the scoping review since his passing. Bryan's commitment to sharing his methods and developing user-friendly tools—paired with his track record of developing new implementation strategies, study designs, measures, and methods—advanced implementation research while making complex concepts more accessible for early career scholars and generalist intervention researchers.

## Conclusion: sustaining impact through enduring relationships

The prior sections briefly summarized Bryan's contributions across five areas of implementation science, all of which addressed the persistent challenge of translating research into real-world practice more rapidly and efficiently. Bryan's work challenged conventional assumptions about the boundaries within research and clinical practice that impede rapid translation of evidence into action. We hope these sections gave the reader a sense of the depth and breadth of his contributions to implementation science. At the same time, we believe that a celebration of Bryan's scientific impact would not be complete without honoring the way he built his teams, cultivated collaborations, and formed enduring friendships.

Bryan was a big tent thinker and a highly inclusive, dynamic leader. He treated every academic conference as an opportunity to meet new colleagues, while strengthening relationships with existing colleagues and friends. He made every scientific event exponentially more fun via his “more the merrier” approach to planning social events. In the same way, he made research meetings more engaging by continually inviting new colleagues and voices to the research study table. Sometimes the groups he created (in research and in social outings) were unexpected and could easily have become unwieldy, but there was always logic to the chaos, and participants would inevitably appreciate Bryan's inclusive approach as they experienced the contribution of diverse thinkers with a wide array of experiences.

At a virtual memorial service for Bryan Garner about a week after his passing, approximately 100 colleagues attended, including his former mentees, colleagues, friends, and community partners. Mentees who had worked with Bryan shared how he consistently asked for, valued, and incorporated their perspectives. Colleagues shared how Bryan challenged them to think bigger and to do more, while making the research process more fun than they thought possible. Organizations that participated in research with Bryan shared that they considered themselves to be true partners, and several partners reflected on how engagement in Bryan's research had a profound impact on them professionally and personally.

Bryan's impact on his teams will endure because he embodied the things he studied. He did not simply study staff turnover and implementation climate; he tirelessly and intentionally cultivated a work climate that encouraged the long-term retention of his staff and that fostered the participation of everyone at the table. Similarly, he did not merely examine how stakeholders made decisions about what to implement in an esoteric or academic way; instead, he engaged stakeholders in decision-making about his own implementation projects from the get-go. He likewise was not satisfied to develop “one-off” pragmatic tools, preferring to disseminate his science in a way that made the novel resources and methods he created accessible to all. His commitment to building a broader and more inclusive tent has inspired many—including the authors of this Editorial—to build upon his exceptional track record of empirical research, continually challenge the status quo in the implementation science field, and support the next generation of implementation scientists.

Bryan's ability to attain academic success, while having such enduring relationships exemplifies a well-known proverb: “*If you want to go fast, go alone. If you want to go far, go together.”* We would all do well to remember this proverb as we seek to honor Bryan's incomparable legacy and support the sustainment of his implementation science mission.
